# The Influence of Antiarrhythmic Device Intervention on Biopsychosocial Functioning and Anxiety in Patients with an Implanted Cardioverter Defibrillator

**DOI:** 10.3390/medicina57020113

**Published:** 2021-01-27

**Authors:** Olimpia Karczewska, Agnieszka Młynarska

**Affiliations:** 1 Department of Anaesthesia and Intensive Nursing Care, Faculty of Health Sciences in Katowice, Medical University of Silesia, 40-635 Katowice, Poland; 2 Department of Gerontology and Geriatric Nursing, Faculty of Health Sciences in Katowice, Medical University of Silesia, 40-635 Katowice, Poland; amlynarska@sum.edu.pl

**Keywords:** implantable cardioverter defibrillator, discharge, concerns, quality of life, anxiety

## Abstract

*Background and objectives:* The aim of the study was to assess the impact of cardioverter-defibrillator interventions on the psychosocial functioning of a patient and the occurrence of concerns related to ICD. *Materials and Methods:* The conducted study was a prospective and observational study that included 158 patients. The study was conducted in two stages: I before ICD implantation and II a follow-up visit six months after the ICD implantation. Standardized questionnaires were used in both stages. *Results:* In the first six months, a cardioverter-defibrillator discharge occurred in 28 participants, which constituted 17.72% of the study group. The number of ICD discharges positively correlated to insomnia, symptoms of anxiety, symptoms of depression, more discharges, more severe insomnia, anxiety and depression. There was also a negative correlation between the number of discharges and the degree of disease acceptance and in the quality of life domains: somatic, mental, social and environmental. The more discharges, the worse the disease acceptance and assessment of the quality of life. *Conclusions:* Individuals who experienced discharges assessed their quality of life as worse in all aspects (perception of the quality of life, own health, physical, mental, social and environmental domains), experienced anxiety and depressive disorders more often, were characterized by a worse functioning in a chronic disease, experienced insomnia more often and reported more concerns related to ICD implantation.

## 1. Introduction

The recommended treatment for patients who are at risk of sudden cardiac death (SCD) is an implantable cardioverter-defibrillator (ICD). Assessing the effectiveness of an ICD in preventing SCD has been and continues to be the subject of many clinical trials. Based on the results, the indications for ICD implantation were developed, which are divided into the indications for secondary prevention—patients after cardiac arrest due to ventricular fibrillation or sustained ventricular tachycardia and the indications for primary prevention—patients without documented malignant arrhythmias, but who are included in the group with a high risk of SCD. Primary prevention is a more complex issue and concerns more patients, including individuals with coronary artery disease, non-ischemic dilated cardiomyopathy, advanced heart failure, hypertrophic cardiomyopathy, arrhythmogenic right ventricular cardiomyopathy, as well as those with long QT syndrome, Brugada syndrome or polymorphic ventricular tachycardia. The criteria for qualification for an ICD is fulfilling the conditions set out in the ESC (European Society of Cardiology) guidelines [1].

Although the benefits of having an ICD are clear, the problems that arise in the psychosocial sphere should also considered. At least 30% of patients exhibit anxiety and depressive symptoms [2]. Patients with an ICD also experience a number of other problems. These include emotional disorders, disturbances in family and intimate relationships, limitations in their physical and professional activity, problems driving a car, poor sleep quality and self-awareness. Patients with greater anxiety related to an ICD are more prone to such disorders. In clinical practice, these disorders are not always diagnosed and treated [2,3,4]. Having a type D personality can be an additional source of problems. An individual with a type D personality is stressed-out or stress-prone individual. Type D patients tend to experience negative emotions such as anger and anxiety, and have a pessimistic approach to life. They react to stress with a sense of helplessness and hopelessness, which can cause many somatic problems (especially cardiac disease). Moreover, cardiological patients who presents type D personality have a worse quality of life, stronger Post Traumatic Stress Disorder (PTSD) symptoms and worse rehabilitation effects [5].

The aim of the study was to assess the impact of cardioverter-defibrillator interventions on the psychosocial functioning of a patient and the occurrence of concerns related to ICD.

## 2. Materials and Methods

### 2.1. Study Design and Setting

The conducted study was a prospective and observational study, which included 158 patients (31 women and 127 men, average age 67.6 ± 9.1), who had been hospitalized for the implantation of an implantable cardioverter-defibrillator at the Department of Electrocardiology and Heart Failure from November 2018 to February 2020. The data to determine the minimum number of individuals within the group was obtained from the EHRA White Book 2017 [6].

### 2.2. Selection of the Study Participants

The study was conducted among 158 patients, who had been hospitalized for the implantation of an implantable cardioverter-defibrillator. The inclusion criteria for the study were eligibility for the ICD implantation procedure in accordance with the ESC guidelines on managing patients with ventricular arrhythmias and preventing sudden cardiac death in 2015, more than 18 years of age and patient consent to participate in the study. Among the exclusion criteria were previously diagnosed mental illness, cancer in the active phase, incomplete documentation of the study, patients with advanced heart failure with indications for treatment with cardiac resynchronization therapy, patients with a previously implanted antiarrhythmic device or not attending the follow-up visit.

All of the patients being treated optimally pharmacologically for cardiovascular causes for a minimum of three months prior to the procedure. The optimal therapy was also continued after the implantation of an ICD. Most of the single chamber ICD devices were programmed as VVIs (VVIRs) at 40 bpm (beats per minute), while the dual chamber devices were programmed as DDDs (DDDRs) at 60 bpm. Sometimes special algorithms were activated according to the manufacturer’s recommendations. Detection programming was also routinely performed according to the manufacturer’s recommendation—the VF area was programmed up to 250 bpm (depending on the device) and the VTs were programmed to about 180 bpm (usually with a 30-interval detection). Activating the discriminator algorithms was usually decided by the doctor who programmed the device.

The study was conducted in two stages.

− Stage I consisted of inclusion in the study before the ICD implantation procedure. In the first stage of the study, a clinical interview was conducted with all of the participants of the study (demographic data, i.e., sex, age, marital status, place of residence, currently used medications, comorbidities), anthropometric measurements were performed and the medical documentation that had been provided by the patient was analyzed. Additionally, standardized questionnaires were completed.
− Stage II included a follow-up visit after six months ± two weeks after ICD implantation. In the second stage of the study six months ± two weeks after the implantation of the ICD, a follow-up visit was conducted, which included a clinical interview regarding the occurrence of any ICD interventions, hospitalizations or complications. In addition, the same questionnaires were completed along with a questionnaire concerning the anxieties of patients with an ICD.

### 2.3. Ethical Considerations

The study protocol was consistent with the provisions of the Helsinki Declaration that was in force at the time of the study. The study was approved by the Bioethics Committee of the Medical University of Silesia in Katowice 30.10.2018 (Resolution KNW/0022/KB/224/I/18). Participation in the study was anonymous and voluntary. Before starting the study, the participants were informed about the confidentiality of the study, its purpose and methodology. The participants were also informed about the possibility to withdraw from the study without having to give any reason. The research was conducted after obtaining the consent of the examined individuals.

### 2.4. Research Instruments

For all patients included in the study, the personality type was assessed using the DS-14 scale. Disease acceptance, quality of life, functioning with a chronic disease, adherence in chronic disease as well as the presence of anxiety, depression or insomnia were also assessed.

The following tools were used in the study:

#### 2.4.1. DS-14 Scale—Scale for Measuring a Type-D Personality

This questionnaire is a tool for examining adults (healthy and sick) that is used to measure the intensity of the stress personality traits (type D). It consists of 14 statements: 7 statements refer to the tendency to experience negative emotions (negative emotionality), while the next 7 statements are about not expressing these emotions and related behaviors (social inhibition). Negative emotionality is the tendency to experience feelings such as anxiety, irritability, anger or dysphoria. Social inhibitions are defined as difficulty in expressing one’s emotions and having social interactions. All of the respondents expressed their opinion about these statements using a 5-point scale: 0—false, 1—rather false, 2—hard to say, 3—rather true and 4—true. Having a type D personality is indicated by a score equal to or greater than ten for both dimensions of a D personality. The higher the score, the greater the intensity of each dimension. Having at least ten points in one of the dimensions indicates an intermediate personality type, while having fewer than ten points in both dimensions means that an individual does not have a D personality. The scale had a high reliability—the Cronbach’s alpha coefficient for the negative emotionality subscale was 0.88, while for social inhibition, it was 0.84 [7].

#### 2.4.2. Acceptance of Illness Scale (AIS)

This is a tool measures the degree of disease acceptance that can be applied to any disease in adults. The scale contains eight statements describing the negative consequences of poor health. These consequences relate to the limitations imposed by a disease, which include a lack of self-sufficiency, a sense of dependence on third parties and a lower self-esteem. All of the AIS statements express the difficulties and limitations that are caused by a disease. A strong agreement with a given statement (grade 1) indicates the non-acceptance of a disease, while a strong disagreement (grade 5) indicates acceptance of a disease. The range of points is between 8 and 40. The higher the value, the better the acceptance, a low value means non-acceptance and a strong sense of psychological discomfort. The sum of the points is used to assess the level of disease acceptance: 8–18 points indicates a lack of acceptance, 19–29 points indicates average level of acceptance and 30–40 points indicates acceptance of the health situation at a good level. The Polish version of AIS has a high Cronbach alpha coefficient—0.82. It is in accordance with original version of the scale [8].

#### 2.4.3. Short Version of the World Health Organization Quality of Life Bref Questionnaire (WHOQOL-Bref)

This questionnaire enables the simultaneous assessment of the quality of life in the somatic, social, psychological and environmental domains of both healthy and sick individuals. The WHOQOL-Bref is a five-point Likert scale. For each question, the respondent can select one of the scored answers. The score within each domain reflects the quality of life in each of these areas. Each item has the same contribution to the score for individual domains (range of points 1–5). Transformation of raw results enables them to be included in a range of 4–20 points, which makes them comparable to the results of the WHOQOL 100. The WHOQOL-Bref instructions were used to convert the raw results into the transformed results. The questionnaire also includes two questions that are analyzed separately. The higher the score for these questions and individual domains, the better the quality of life. According to the definition of the WHO BREF questionnaire, a time frame of two weeks is indicated in the assessment [9]. The Cronbach’s alpha coefficient reflecting the reliability of the instrument for the total sample was satisfactory (for somatic, psychological and environmental domains, reliability coefficients were higher than 0.70, the social domain exhibited a lower value of 0.69) [10].

#### 2.4.4. Hospital Anxiety Depression Scale (HADS)

The HADS scale for assessingis used to assess anxiety and depression;—the HADS scale (Hospital Anxiety Depression Scale) is a commonly used tool for assessing anxiety and depression, which has been adapted to patients suffering from various somatic diseases. Its objective is to assess the patient’s negative emotions. The scale measures a condition, not a feature. It is characterized by its high sensitivity and specificity. The internal consistency, measured as Cronbach’s alpha, showed optimal results for both subscales and for the whole questionnaire (0.88) [11]. The scale consists of seven statements relating to anxiety and seven statements relating to depression, which can be rated from 0 to 3 points. The lower the score, the lower the severity of the disorder. A score from 0–7 points indicates no disorders, 8–10 points indicates a borderline condition and 11–21 points is characteristic of mood disorders [12].

#### 2.4.5. The Functioning in the Chronic Illness Scale (FCIS)

The scale is used to identify deficit areas in the functioning of a patient with a chronic disease, which is necessary to determine the appropriate therapeutic measures. The questionnaire assesses the impact of a disease on the patient, the patient’s impact on a disease and the impact of a disease on the patient’s attitude. The first part of the questionnaire is the patient’s subjective assessment of how a disease affects their life. The second part of the questionnaire specifies the patient’s views on the possibility of impact on he course of the disease. A maximum score indicates that a patient feels that they have a significant impact on the course of a disease. While a minimum score indicates that a patient believes that they have no impact on the course of a disease. The third part describes the patient’s attitude towards a new life situation. A maximum score indicates that a patient is very optimistic about the future, whereas the minimum score indicates that they see the future rather pessimistically. The result is the sum of the first, second and third subscales. The maximum score indicates that a patient is functioning very well with a disease, while the minimum indicates that a patient is functioning poorly. The internal consistency of the questionnaire, which is expressed with Cronbach’s alpha coefficient = 0.855, indicates its high reliability and homogeneity. The validation procedure revealed that the FCIS is a reliable and homogeneous tool for measuring the physical and mental functioning of a patient with a chronic disease [13].

#### 2.4.6. The Adherence in Chronic Diseases Scale (ACDS)

The ACDS is a tool for evaluating the execution of a specific treatment plan. The questionnaire contains seven questions that have five suggested answers for each question (the answers are successively scored A—4 points, B—3 points, C—2 points, D—1 point and E—0 points). Those questions include the behavior that directly determines compliance with the rules (questions 1–5), as well as situations and views that may indirectly affect such compliance (questions 6 and 7). The number of points that is obtained indicates the percentile norms: ≤20 points—low level, 21–26 points—medium level and ≥27 points—high level. The ACDS is intended to evaluate adults who are being treated for a chronic disease. The tool not only reflects the implementation of the pharmacological treatment plan, but also identifies the mechanisms that impact compliance with the rules. The patient’s understanding of the sense of the diagnostic and therapeutic measures fosters their acceptance and substantially improves the effectiveness of the therapy. The scale was validated in the group 413 patients with coronary heart disease. The Adherence Scale in Chronic Diseases is a consistent and well validated instrument for identifying specific obstacles to medication adherence [14].

#### 2.4.7. The Athens Insomnia Scale (AIS)

This scale enables the quantitative measurement of insomnia symptoms on the basis of the ICD-10 criteria. The original validation studies demonstrated the high reliability and validity of this tool. It is a self-reporting tool that consists of eight statements concerning various symptoms of insomnia. Each item is assessed by the respondent on a scale of 0–3 points, where 0 indicates no symptoms, while 3 indicates it’s a symptom’s significant intensity. The total score on the scale is in the range of 0–24 points. A score of six or more points is considered the value that enables a high probability of insomnia to be confirmed (scale sensitivity—93%, scale specificity—85%). It is one of the most frequently used scales for both diagnostic purposes and in research on the effectiveness of insomnia treatments [15].

#### 2.4.8. The ICD Patient Concerns Questionnaire (ICDC)

This questionnaire contains 20 questions that are scored on a 5-point Likert scale: from 0—not applicable to 4 points, which corresponds with a high intensity. The points range from 0 to 20 (for the number of concerns) and from 0 to 80 (increased concern). The number of fears and the severity these fears can also be combined in order to obtain the total number concerns (maximum 100). The scale also has two subscales—factor 1 assesses the perceived limitations and factor 2 assesses device specific concerns. A higher result indicates a greater severity of a concern. The Cronbach alpha that characterized the internal consistency of the entire questionnaire was 0.96. [16,17].

### 2.5. Statistical Analyses

The quantitative variables were analyzed by calculating the mean, standard deviation, median, quartiles, minimum and maximum. The analysis of qualitative variables were analyzed by calculating the number and percentage of the occurrences of each value. The analysis of the survey questions were analyzed by calculating the number and percentage of the occurrences of each answer. The values of quantitative variables in two groups were compared using the Mann-Whitney test. A significance level of 0.05 was adopted in the analysis. Thus, all *p* values below 0.05 were interpreted as showing significant dependencies. The Spearman correlation coefficient r was used to correlate the concern about an ICD implantation and insomnia, the symptoms of anxiety and depression and the number of discharges. The analysis was performed in the R software, version 4.0 (R Core Team, Vienna, Austria) [18].

## 3. Results

All of the patients who had qualified for the study reported for a follow-up visit (80.38% of the men and 19.62% of the women). Most of the patients lived in a city, i.e., 82.91%. Individuals in a relationship accounted for 70.25% and single individuals for 29.75% of the study group. As many as 91% were individuals who had children and while 83.5% of the respondents lived with their immediate family, 16.5% lived alone. The vast majority denied the use of stimulants such as cigarettes (76.5%) and alcohol (97.5%). Most of the patients who had qualified for implantation of an ICD had the indication for primary prevention—85.44%. Patients with the indication for secondary prevention accounted for 14.56%. Single-chamber cardioverter-defibrillators—VVIs were implanted in 38.6% of the patients, dual-chamber cardioverter-defibrillators DDDs were implanted in 61.4% of the patients and no patients had an SICD implanted.

Detailed characteristics of the study group are presented in Table 1.

### Follow up

All of the patients included in the study reported for a follow-up visit. In the first six months, 28 individuals experienced a cardioverter-defibrillator discharge, which constituted 17.72% of the study group. Among those patients, 85.71% were men and 14.28% were women (*p* = 0.6021). During the follow-up period, 86% patients developed ATP (antitachycardia pacing), however, none of the patients underwent cardioversion nor was there any direct current (DC) shocks.

**Figure 1:**
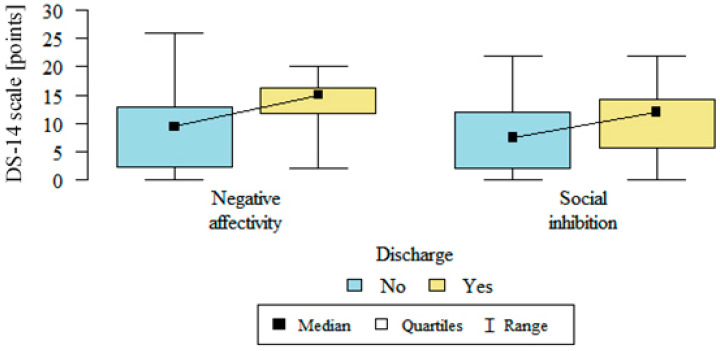
Negative affectivity and social inhibition in patients with and without a discharge (DS-14 scale—scale for measuring a type-D personality, no—without a discharge, yes—with a discharge)**.**

Individuals with type D personality accounted for 39.87% of the participants, individuals with an intermediate personality type accounted for 23.41% and rest, i.e., 36.7%, were individuals with a non-D type personality. In the group with no discharges, negative emotionality was on average 8.61 ± 6.21 and social inhibition was 7.68 ± 5.62. Both negative emotionality (13.54 ± 4.62; *p* <0.001) and social inhibition (11.18 ± 6.2; *p* = 0.006) were stronger in the group with defibrillators that had discharged. Details are presented in Figure 1.

**Figure 2:**
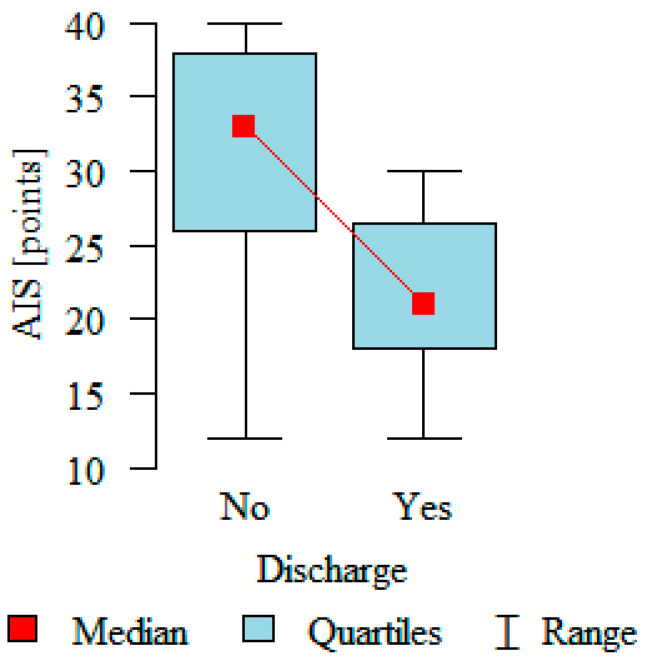
The disease acceptance after ICD implantation in patients with and without a discharge (AIS-Acceptance of Illness Scale, no—without a discharge, yes—with a discharge).

The disease acceptance (AIS) after implantation showed a statistically significant dependency (*p* < 0.05). The disease acceptance level was significantly higher in the group with no discharges and was 31.15 ± 7.7; in the group with defibrillators that had discharged, the level reached 21.64 ± 5.39; *p* < 0.001. Details are presented in Figure 2.

**Figure 3:**
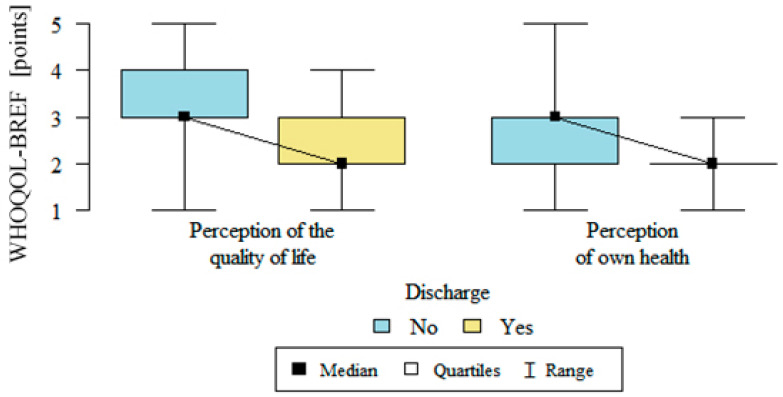
Perception of quality of life and perception of own health after ICD implantation in patients with and without discharge (no—without a discharge, yes—with a discharge).

**Figure 4:**
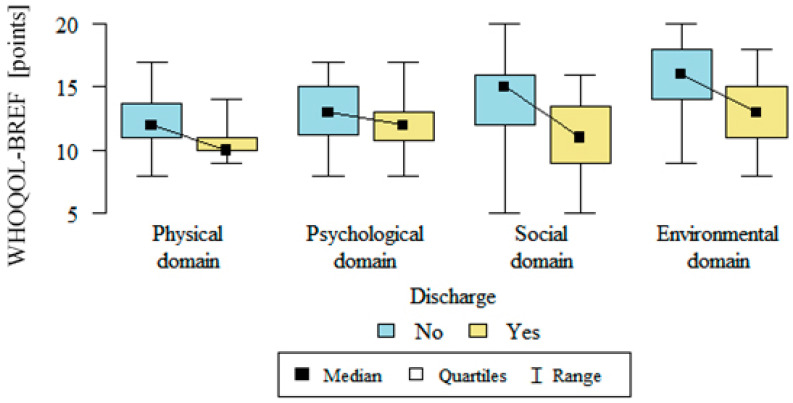
Quality of life in the physical, psychological, social and environmental domains after ICD implantation in patients with and without discharge (WHOQOL Bref- World Health Organization Quality of Life Bref questionnaire, no—without discharge, yes—with discharge).

The quality of life, which was assessed using the WHOQOL-BREF questionnaire, was significantly lower in all of the domains for all of the respondents. After implantation, there was statistically significant relationship: the quality of life in all of the domains was significantly better in the group with defibrillators that had not discharged. Details are presented in Table 2, Figure 3 and Figure 4.

**Figure 5:**
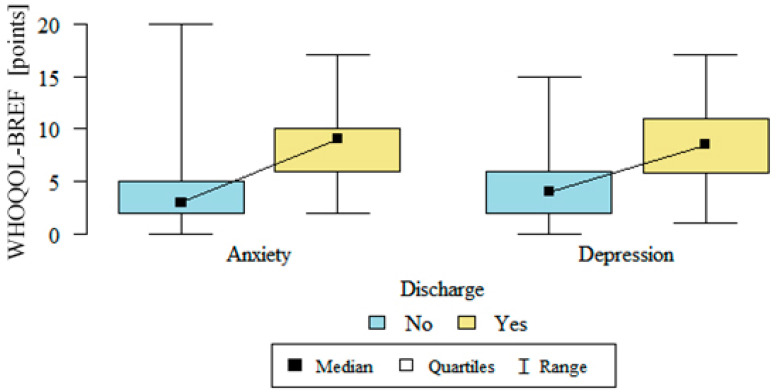
Anxiety and depression after ICD implantation in patients with and without discharge (WHOQOL Bref-World Health Organization Quality of Life Bref questionnaire, no—without a discharge, yes—with a discharge).

The severity of anxiety and depression after implantation, which was assessed using the HADS scale, was statistically significantly higher in the group with defibrillators that had discharged, than in patients with no discharges. The level of anxiety in the group with discharges was on average 8.21 ± 3.07 vs. 3.79 ± 2.85 in the non-discharge group; *p* < 0.001; the depression level was on average 8.39 ± 3.92 in the group with defibrillators that had discharged vs. 4.68 ± 3.39 in the group without discharges, *p* < 0.001. Details are presented in Figure 5.

Functioning with a chronic disease after implantation, which was assessed using the FCIS questionnaire, also showed a statistically significant dependence. Patients who did not experience cardioverter-defibrillator discharges performed statistically significantly better in the parts concerning the impact of the disease on the patient, the patient’s impact on the disease and the disease’s impact on the patient’s attitudes. Details are presented in Table 3.

**Figure 6:**
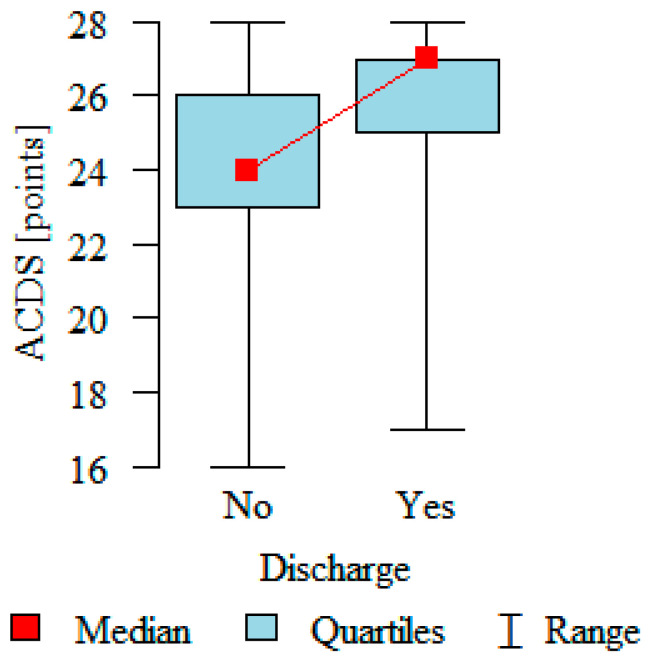
Adherence in patients with a chronic disease after ICD implantation with and without discharge (ACDS-Adherence in Chronic Disease Scale, no—without a discharge, yes—with a discharge).

Adherence in the group with no defibrillator discharges was 24.25 ± 2.43 on average; while in the group with discharges, the average adherence was 25.71 ± 2.59; *p* < 0.001 was statistically significantly higher. Patients who experienced discharges adhered to the therapeutic recommendations better. All of the patients enrolled in the study demonstrated moderate adherence. Details are presented in Figure 6.

Insomnia after implantation, which was assessed using the Athenian Insomnia Scale also showed a statistically significant dependence. Insomnia was less severe in the group with no defibrillator discharges—on average of 4.1 ± 4.38. It was significantly more intense in the group with discharges and was 7.07 ± 4.83, *p* < 0.001 on average.

Patient concerns after the implantation of an ICD were assessed using the ICDC questionnaire. All of the elements of the scale showed statistically significant relationships. The number of concerns, their severity, overall concerns score, factor 1—overall concerns score and factor 2—overall concerns score were significantly higher in the group with defibrillator discharges. Details are presented in the Table 4.

An analysis of the impact of the number of discharges of the implanted device on patient concerns showed that there was a positive correlation between the number of discharges and the number of concerns (*r* = 0.541; *p* < 0.001), the intensity of the concerns (*r* = 0.611; *p* < 0.001), the overall concerns score (*r* = 0.602; *p* < 0.001), factor 1—overall concerns score (*r* = 0.606; *p* < 0.001) and factor 2—overall concerns score (*r* = 0.596; *p* < 0.001). The number of discharges statistically significantly correlated with the severity of anxiety and fear in the patients with ICD.

The number of ICD discharges positively correlates to insomnia (*r* = 0.285; *p* < 0.001), the symptoms of anxiety (*r* = 0.493; *p* < 0.001), the symptoms of depression (*r* = 0.360; *p* < 0.001); the more discharges, the greater the severity of insomnia, anxiety and depression. There was also a negative correlation between the number of discharges and the degree of disease acceptance (*r* = −0.42, *p* < 0.001) and quality of life domains: somatic (*r* = −0.428; *p* < 0.001), mental (*r* = −0.260; *p* < 0.001), social (*r* = −0.317, *p* < 0.001) and environmental (*r* = −0.324; *p* < 0.001). The more discharges, the worse the disease acceptance and assessment of the quality of life.

## 4. Discussion

The patient population with an implanted cardioverter defibrillator is a very specific population. The results of the conducted study indicate that patients with an implanted cardioverter-defibrillator have to deal with anxiety, which were more severe in the group of patients who experienced a defibrillator discharge in the first six months after implantation. A study by Pedersen et al. concerning anxiety disorders in patients with an ICD examined the relative importance of the experienced discharges compared to the subjective concerns about an ICD as determinants of anxiety, fear and depression [2]. In our study, 85.44% of the patients that had an ICD were in the primary prevention group and 14.56% were in the secondary prevention group. Our results are consistent with the results of the ADVANCE III study, in which implantations in the secondary prevention patients accounted for 25% of the procedures [19]. In other studies, there were lower rates of primary prevention (PainFREE II implantations in primary prevention accounted for 48% of the implantations [20] and in the OPERA registry only 30% [21]); however, the study inclusion criteria were different, and were often dependent on the sponsor of the study, which was often the device manufacturer. Our study included consecutive patients in the period being studied.

An ICD discharge in the above-mentioned study occurred in 30% the patients, and the individuals who experienced them had higher scores regarding the concerns in the ICDC questionnaire, which was also used in our study. In a study by Pedersen et al., 32% of patients experienced anxiety, while in 28% experienced depressive symptoms. Both symptoms were assessed using the HADS questionnaire and were more common for patients who had expressed a high number of concerns in the ICDC [2]. Similarly, the present study found higher levels of anxiety and depressive symptoms among the patients who had experienced a device intervention.

In a study by Denollet et al., the prognostic significance of a type D personality and discharges in patients with an ICD was assessed. Type D personality was determined in 23% of the respondents. It was also shown that both the occurrence of discharges and the presence of type D personality were associated with an increased risk of mortality [22]. In our study, type D personality was determined in 39.8% of the respondents, and occurred significantly more often in the group with discharges. The difference in the prevalence of type D personality in the previous studies may have resulted from the difference in the size of the groups in the study by Denollet et al. in which 589 individuals were examined, while there were only 158 individuals enrolled in the present study. The literature states that there is a possibility of personality changes in people at risk of experiencing high and long-term stress. However, this appears to require a longer exposure time of at least two years [23,24].

A good quality of life is also an extremely important factor when treating patients. Hoogwegt et al. examined the stress of patients after ICD implantation using a proprietary questionnaire [3]. In the present study, a standardized tool, the WHOQOL-Bref questionnaire, was used to examine the quality of life. Quality of life was rated as being worse in all of the domains in the group of patients who had experienced defibrillator discharges. In a study by Jacq et al., the level of anxiety, depression and quality of life were assessed in two groups of patients—patients with a discharge and with no discharge. The level of anxiety did not differ significantly in the two groups. On the other hand, the depressive symptoms were significantly higher in the group that had experienced discharges. The psychological aspects of the quality of life were also lower in the group with discharges [25]. In our study, similar to the study by Jacq et al., the psychological aspects of the quality of life were perceived to be worse, and the symptoms of depression were significantly higher in the group that had experienced discharges. In addition, our study showed that all of the other areas that constitute a good quality of life such as the perception of one’s health, physical, social and environmental domains were not assessed highly in the group of patients who experienced defibrillator discharges. Most studies have confirmed the negative effect of ICD discharge on the patient’s quality of life. Of course, an adequate discharge saves the patient’s life, however, in the authors’ opinion, the patient usually does not know whether the shock was adequate or not, but the only thing he can say is the negative feelings associated with the intervention. The impact on the quality of life is an individual variable. However, other factors, including comorbidities, also influence the assessment of quality of life [26,27,28].

Patients with an implanted cardioverter defibrillator were also diagnosed with comorbidities. The most numerous diseases in this group were arterial hypertension (81.6%), ischemic heart disease (77.8%) and diabetes (42.4%). Similar populations of patients were included in the PainFREE II study (ischemic disease was found in 84% of patients, arterial hypertension in 56%) [20] and in the ADVENCE III study, where 59% of patients had ischemic disease [19].

An important factor in the functioning of patients who undergo ICD implantation is the early detection of psychological distress. The aim of a cohort study by Versteeg et al. was to assess the frequency and markers of psychological distress in patients after ICD implantation early. The incidence of anxiety was 16%, depression was 19%, and 25% of patients reported one or both disorders in the first two weeks after implantation [29]. In the present study, the evaluation was performed six months after implantation. Anxiety and depressive disorders were significantly more frequent in the group of patients that had experienced defibrillator discharges. Perhaps, these disorders should be assessed twice, in the early period after implantation (two weeks) and six months after the procedure. Identifying patients with psychological distress early would enable cardiac rehabilitation and psychological care to be introduced, which, by reducing anxiety and depressive disorders, would limit the inadequate intervention of a device. In a study by Kamphuis et al., similar to our study, the quality of life of patients after ICD implantation was assessed as well as the presence of anxiety and depression in the group with and with no discharges. Patients were assessed 1, 6 and 12 months after implantation. Twenty-six percent of the patients experienced defibrillator discharges (usually in the last six months of the study). The quality of life of these patients did not change significantly during the first six months. Anxiety and depressive disorders were significantly more common in the individuals who had experienced a discharge. It was shown that these individuals did not adapt well after ICD implantation [30]. Similarly, our study showed that in the group that had experienced discharges, the quality of life deteriorated, and the level of anxiety and depression was significantly higher. Our study, however, was focused on a six-month follow-up. A number of new questionnaires concerning the functioning of patients with an ICD were used in this research. Defibrillator cardioverter discharges are associated with concerns about and ICD and affect a number of patient behaviors, including treatment adherence, disease acceptance and chronic disease functioning. The questionnaire that was used to assess the concerns of an ICD patient is a new tool in the national language version.

The results of our work confirm the necessity of implementing psychological care in patients with an implanted cardioverter defibrillator, and in particular, in patients who have experienced defibrillator cardioverter discharges. We are aware that not all patients will need psychological help, however, even the awareness of the possibility of receiving support can be extremely important for a patient.

### Study Limitation

The study was a single center study and the patients that were included in the study were from one region. Moreover, only a relatively small group of patients was included in the study. The occurrence of a discharge, regardless of whether it is adequate or inadequate, is the same experience and stress for the patient, therefore we did not analyse the adequacy of the discharge.

## 5. Conclusions

In the first 6 months after ICD implantation, almost one fifth of the patients experienced defibrillator discharges. Individuals who had experienced discharges assessed their quality of life as being worse in all aspects (perception of the quality of life, own health, physical, mental, social and environmental domains), experienced anxiety and depressive disorders more often, were characterized by worse functioning in a chronic disease, experienced insomnia more often and reported more concerns related to The ICD implantation. Patients with cardioverter defibrillator discharges demonstrated better adherence.

## Figures and Tables

**Table 1:** Characteristics of the patients included in the study.

Parameter		All (*n* = 158)
Age (years)	average ± SD	67.61 ± 9.15
	median	68
	quartile	62–74
BMI (kg/m^2^)	average ± SD	29.01 ± 5.84
	median	28.71
	quartile	24.82–31.77
SBP (mmHg)	average ± SD	123.32 ± 12.87
	median	125
	quartile	110–130
DBP (mmHg)	average ± SD	74.8 ± 8.93
	median	75
	quartile	70–80
EF (%)	average ± SD	29.75 ± 8.34
	median	29
	quartile	25–33
Marital status	Single	47 (29.7%)
	Married/living with partner	111 (70.2%)
Education	None or primary	6 (3.8%)
	Vocational	93 (58.9%)
	Secondary	41 (25.9%)
	Higher	18 (11.4%)
Disease	Heart Failure (NYHA I–IV)	140 (88.6%)
	Hypertension	129 (81.6%)
	Stroke	14 (8.8%)
	Ischemic Heart Disease	123 (77.8%)
	COPD	18 (11.3%)
	Obesity	71 (44.9%)
	Thyroid Disease	20 (12.6%)
	Diabetes	67 (42.4%)
Medications	β blocker	156 (98.1%)
	ACE	144 (91.1%)
	MRA	147 (93%)
	Statins	136 (86%)
	Oral anti-diabetic drugs	63 (39.8%)
	Insulin	26 (16.45%)

SD-Standard Deviation, BMI-Body Mass Index, SBP-systolic blood pressure, DBP-diastolic blood pressure, EF-Ejection Fraction, NYHA-New York Heart Association, COPD—Chronic Obstructive Pulmonary Disease, ACE—Angiotensin-converting enzyme inhibitors, MRA—Mineralocorticoid receptor antagonists.

**Table 2:** Quality of life of patients after ICD implantation.

WHOQOL BREF		Discharge		*p*
		No (*n* = 130)	Yes (*n* = 28)	
Perception of quality of life	average ± SD	3.31 ± 0.85	2.43 ± 0.63	*p* < 0.001 *
	median	3	2	
	quartile	3–4	2–3	
Perception of own health	average ± SD	2.64 ± 0.77	1.86 ± 0.45	*p* < 0.001 *
	median	3	2	
	quartile	2–3	2–2	
Physical domain	average ± SD	12.18 ± 1.9	10.71 ± 1.41	*p* < 0.001 *
	median	12	10	
	quartile	11–13.75	10–11	
Psychological domain	average ± SD	13.21 ± 1.96	11.71 ± 2.16	*p* = 0.001 *
	median	13	12	
	quartile	11.25–15	10.75–13	
Social domain	average ± SD	14.28 ± 2.86	11.43 ± 3.19	*p* < 0.001 *
	median	15	11	
	quartile	12–16	9–13.5	
Environmental domain	average ± SD	15.6 ± 2.54	13.14 ± 2.69	*p* < 0.001 *
	median	16	13	
	quartile	14–18	11–15	

*p*—Mann-Whitney test, * statistically significant relationship (*p* < 0.05), SD—Standard Deviation, WHOQOL Bref—World Health Organization Quality of Life Bref questionnaire, no—without discharge, yes—with discharge.

**Table 3:** Functioning with a chronic disease in patients after ICD implantation.

FCIS		Discharge		*p*
		No (*n* = 130)	Yes (*n* = 28)	
Part I—The impact of the illness on the patient	average ± SD	24.83 ± 6.67	17.61 ± 5.14	*p* < 0.001 *
	median	25	16	
	quartile	20.25–29	14–21	
Part II—The patient’s impact on the illness	average ± SD	26.23 ± 4.13	22.71 ± 3.09	*p* < 0.001 *
	median	26	23	
	quartile	24–28	20–25	
Part III—The impact of the illness on patient’s attitude	average ± SD	28.19 ± 4.98	22 ± 4.26	*p* < 0.001 *
	median	28	21	
	quartile	25–31.75	19–24.25	
Total FCIS	average ± SD	79.28 ± 14.59	62.75 ± 11.03	*p* < 0.001 *
	median	79	60	
	quartile	69.25–91	53.75–70	

*p*—Mann-Whitney test, * statistically significant relationship (*p* < 0.05), SD-Standard Deviation, FCIS—Functioning with a chronic disease no-without discharge, yes-with discharge.

**Table 4:** Severity of concerns in patients after ICD implantation.

ICDC		Discharge		*p*
		No (*n* = 130)	Yes (*n* = 28)	
Number of concerns	average ± SD	6.29 ± 4.32	13.5 ± 3.13	*p* < 0.001 *
	median	6	13.5	
	quartile	3.25–9	11–16	
Intensity of concerns	average ± SD	9.7 ± 9.43	36.86 ± 10.76	*p* < 0.001 *
	median	6	36.5	
	quartile	4–14	28–44.25	
Overall concerns score	average ± SD	15.99 ± 13.33	50.36 ± 13.49	*p* < 0.001 *
	median	12	49	
	quartile	6.5–22.75	41.5–60	
Factor 1—perceived limitations	average ± SD	2.93 ± 3.6	14.5 ± 6.7	*p* < 0.001 *
	median	2	14	
	quartile	0–4	9.75–20	
Factor 2—device specific concerns	average ± SD	5.27 ± 5.57	19.54 ± 5.51	*p* < 0.001 *
	median	3	20	
	quartile	2–6.75	15.5–23	

*p*—Mann-Whitney test, * statistically significant relationship (*p* < 0.05), SD-Standard Deviation, ICDC-Implantable Cardioverter Defibrillator Concerns Questionnaire, no-without discharge, yes-with discharge.

## Data Availability

Data sharing not applicable.
